# Hepatitis B Virus Reactivation under Ibrutinib Treatment in a Patient with Chronic Lymphocytic Leukemia

**DOI:** 10.4274/tjh.galenos.2020.2019.0180

**Published:** 2020-08-28

**Authors:** Gülşen İskender, Dicle İskender, Mustafa Ertek

**Affiliations:** 1 Dr. Abdurrahman Yurtaslan Ankara Oncology Training and Research Hospital, Clinic of Infectious Diseases and Clinical Microbiology, Ankara, Turkey; 2 Dr. Abdurrahman Yurtaslan Ankara Oncology Training and Research Hospital, Clinic of Hematology and Blood and Marrow Transplantation, Ankara, Turkey

**Keywords:** Hepatitis B reactivation, Ibrutinib, Chronic lymphocytic leukemia

## To the Editor,

**Figure 1:**
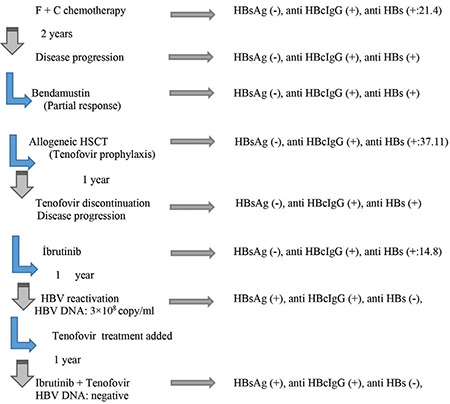
Summarized course of the disease. HBsAg: Hepatitis B surface antigen.

Immunosuppression in patients with hepatitis B virus (HBV) infection may result in viral reactivation. This risk is higher in patients with present than past HBV infection (hepatitis B surface antigen (HBsAg)-positive vs. HBsAg-negative, anti-HBc positive) and in patients with hematological malignancies and related treatments [1,2]. Ibrutinib is a Bruton’s tyrosine kinase inhibitor (TKI) indicated in the treatment of relapsed/refractory chronic lymphocytic leukemia (CLL) [3]. The American Gastroenterological Association Institute categorized patients with past HBV infection treated with TKIs as having a moderate risk for HBV reactivation (HBVr) (1%-10%). There is only a weak recommendation for routine viral prophylaxis for HBVr in that guideline [2], whereas in the ECIL-5 guideline, there is no suggestion about the management of these patients [4]. Here we describe a case of HBVr under ibrutinib monotherapy in a patient with past HBV infection and relapsed/refractory CLL. A 58-year-old man was diagnosed with CLL. His HBV serology was compatible with past infection (Figure 1). According to existing guidelines [1,2], follow-up of liver enzymes, HBV serology, and HBV-DNA every 3 months was planned without antiviral prophylaxis. Following first-line treatment with fludarabine and cyclophosphamide, the disease progressed, and bendamustine was started. He achieved a good partial response and allogeneic hematopoietic stem cell transplantation (HSCT) was performed under antiviral prophylaxis by tenofovir disoproxil at 245 mg/day. HBV serology prior to HSCT was as follows: HBsAg (-), anti-HBc IgG (+), anti-HBs (+). Antiviral prophylaxis was discontinued 1 year after HSCT but disease progression was detected and ibrutinib was started at 420 mg/day. At this time, the serological results did not change (Figure 1). One year later while he was still on ibrutinib treatment, HBV serology showed reactivation with HBsAg (+), anti-HBc IgG (+), anti-HBs (-), HBeAg (+), and HBV DNA of 3x10^3 ^copies/mL that progressed to 1x10^8 ^copies/mL over 30 days, with normal liver enzymes. Treatment with tenofovir disoproxil at 245 mg/day was therefore initiated. HBV DNA levels decreased progressively and reached undetectable levels within 1 year. At the time of this report, he is on ibrutinib and tenofovir treatment with HBsAg (+), anti-HBc IgG (+), anti-HBs (-), HBeAg (+), and negative HBV DNA. The course of the disease is summarized in Figure 1.

Ibrutinib has B-cell signaling inhibitory activity that might be more potent than anti-CD20 monoclonal antibodies. HBVr in the case of chronic HBV carrier state or past HBV infection could be a potential complication of treatment with this agent [5]. In our patient, occurrence of HBVr 2 years after allogeneic HSCT and 1 year after ibrutinib makes ibrutinib the possible cause of reactivation. Because HBsAg positivity was considered as an exclusion criterion in ibrutinib clinical trials, no recommendation was provided in guidelines for the management of HBsAg (-)/anti-HBc (+) patients who are being treated with this agent [6]. Reports have been published since 2015 that emphasize the effect of ibrutinib on HBVr in patients with past HBV infection [5,7,8]. These reports along with our case emphasize the risk of HBVr secondary to ibrutinib use in HBsAg (-), anti-HBc (+) patients. In our opinion, there is a need for an international consensus to support the recommendation of antiviral prophylaxis against HBVr in this group of patients.
